# Ar-DAD: Arabic diversified audio dataset

**DOI:** 10.1016/j.dib.2020.106503

**Published:** 2020-11-07

**Authors:** Mohammed Lataifeh, Ashraf Elnagar

**Affiliations:** Department of Computer Science, University of Sharjah, Sharjah 27272, United Arab Emirates

**Keywords:** Arabic audio clips, Speaker identification, Machine learning, Deep learning, Quran recitations, Imitators, Cantillations

## Abstract

The automatic identification and verification of speakers through representative audio continue to gain the attention of many researchers with diverse domains of applications. Despite this diversity, the availability of classified and categorized multi-purpose Arabic audio libraries is scarce. Therefore, we introduce a large Arabic-based audio clips dataset (15810 clips) of 30 popular reciters cantillating 37 chapters from the Holy Quran. These chapters have a variable number of verses saved to different subsequent folders, where each verse is allocated one folder containing 30 audio clips for the declared reciters covering the same textual content. An additional 397 audio clips for 12 competent imitators of the top reciters are collected based on popularity and number of views/downloads to allow for cross-comparison of text, reciters, and authenticity. Based on the volume, quality, and rich diversity of this dataset we anticipate a wide range of deployments for speaker identification, in addition to setting a new direction for the structure and organization of similar large audio clips dataset.

## Specifications Table

**Table:** 

Subject	Computer Science
Specific subject area	Speaker identification; imitation detection
Type of data	Audio files
	Plain text files
How data were acquired	Scrapping and extracting WAV audio files from a Quranic audio portal.
Data format	Raw
	Filtered
Parameters for	data
collection	The main reciters’ data were collected from a Quranic audio portal. The audio data of 30 reciters covering chapters 78-114 were scrapped in WAV format. The textual contents of these chapters were also collected in plain text, with and without vocalization (vowelization or Tashkeel in Arabic).
	Additionally, 12 popular imitators were identified based on social media popularity, and a total of 379 imitations audio clips were collected from multiple websites.
Description of data collection	The dataset is organized into three different directories. Reciters directory contains (37 folders, 15,810 files) the audio files organized by Chapter> Verse > Reciter covering all Quranic chapters from 78 to 114. The extracted audio files are enumerated with codes corresponding to each reciter.
	The second directory includes two plain text files that cover the textual materials for the same chapters with and without vocalization.
	The Imitators’ directory includes 379 audio clips for 12 anonymous highly skilled imitators identifies by a prefix ID and listed in one single folder. The imitations included verses from chapters other than the ones covered in the reciters directory to allow for text-independent analysis.
Data source location	Islamic endowment site for Holy Quran recitations >http://www.everyayah.com
Data accessibility	The Data is made available on Mendeley Data under Creative Commons Attribution 4.0 International, publicly available via the link: https://doi.org/10.17632/3kndp5vs6b.3
Related research article	M. Lataifeh, A. Elnagar, I. Shahin, and A. Bou Nasif, Arabic audio clips: Identification and discrimination of authentic Cantillations from imitations, Neurocomputing, 418 (2020) 162177. https://doi.org/10.1016/j.neucom.2020.07.099.

## Value of the Data

• To the best of our knowledge, this repository is the only available and representative dataset for the Arabic Audio clips performed by 30 popular reciters from different countries.
• An additional subset of 397 audio clips for imitators of top reciters.
• The dataset paves the road for further research on speech and speaker recognition as well as the recognition and identification of genuine/imitation sound clips of a speaker/reciter.
• The scale and accessibility of data format enable researchers to implement classical machine learning and deep learning classifiers.

## 1 Data Description

### 1.1 Dataset

The audio clips dataset described here is the only available audio library covering this large number of popular reciters and verses in one harmonized structure that can be used for a variety of applications. The Audio dataset is composed of two subsets identified as reciters and imitators in addition to a third directory that contains the plain text materials for the same range of verses.

The dataset is organized into three different directories. Reciters’ directory contains (37 folders, 15,810 files) the audio files organized by Chapter> Verse > Reciter covering all Quranic chapters from 78 to 114. Each chapter has several subfolders enumerated by the number of the verse. Each verse folder contains 30 audio clips, one for each of the mentioned reciters cantillating the same verse.

**Fig. 1:**
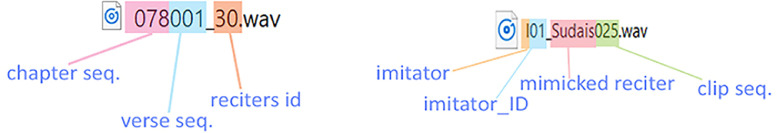
Reciters and imitators audio clip file name details.

The Imitators’ directory includes 379 audio files covering 12 anonymous highly skilled imitators identified by a prefix ID and listed in one single folder structure to host all audio clips. The files can be identified through the file name starting with an "I" as a reference to the imitators, followed by an imitator ID sequence, the reciters’ name being imitated, and finally a sequence number for the clip in series of other clips covering the same reciter, but not necessarily the same textual content which adds to the advantages of the dataset as it supports both text-dependent and text-independent evaluations. See Fig. 1 for more details on clips naming convention.

The third directory simply includes two plain text files that cover the textual materials for the same chapters covered by the reciters with and without vocalization (vowelization).

The data is shared as WAV format with the common sampling rate of 44.1 kHz, 16 Bit depth, and Stereo Channels as they were disseminated over the internet as an Islamic endowment materials [5], which is very common in the Islamic World where hundreds other entities share similar materials provided in different format for basic recitation, learning, or memorization of the Holy Quran all together. Both manual and automatic approaches were used in scrapping the audio clips (with no further enhancements) to ensure data consistency for the reciters’ audio clips in the WAV format as being largely supported by several machine learning framework.

### 1.2 Data Extraction

All files have been compressed into a series of smaller zipped parts to facilitate the download of such large directory in a single compressed archive, which could not be uploaded as well for exceeding the maximum size allowed by Mendeley as a host for the dataset. Once the series of these files are downloaded, extract the compressed parts using the file named “Audio_dataset.zip” which will verify all necessary sequential parts and create the described directories of the dataset. If any error is faced during extraction, the extractor will specify the corrupted part, instead of having to download all the compressed parts again.

The second dataset includes only one folder of audio clips for imitators categorized by an anonymous imitator identification number. Twelve imitators were selected based on popularity and number of views that range on YouTube for instance from hundreds of thousands to millions. Imitators differed in popularity according to their mastery level in imitating or mimicking the melodic style of a reciter, which explains the imbalanced data coverage as it was difficult to collect equal representative audio clips as carried out in the reciters dataset.

**Fig. 2:**
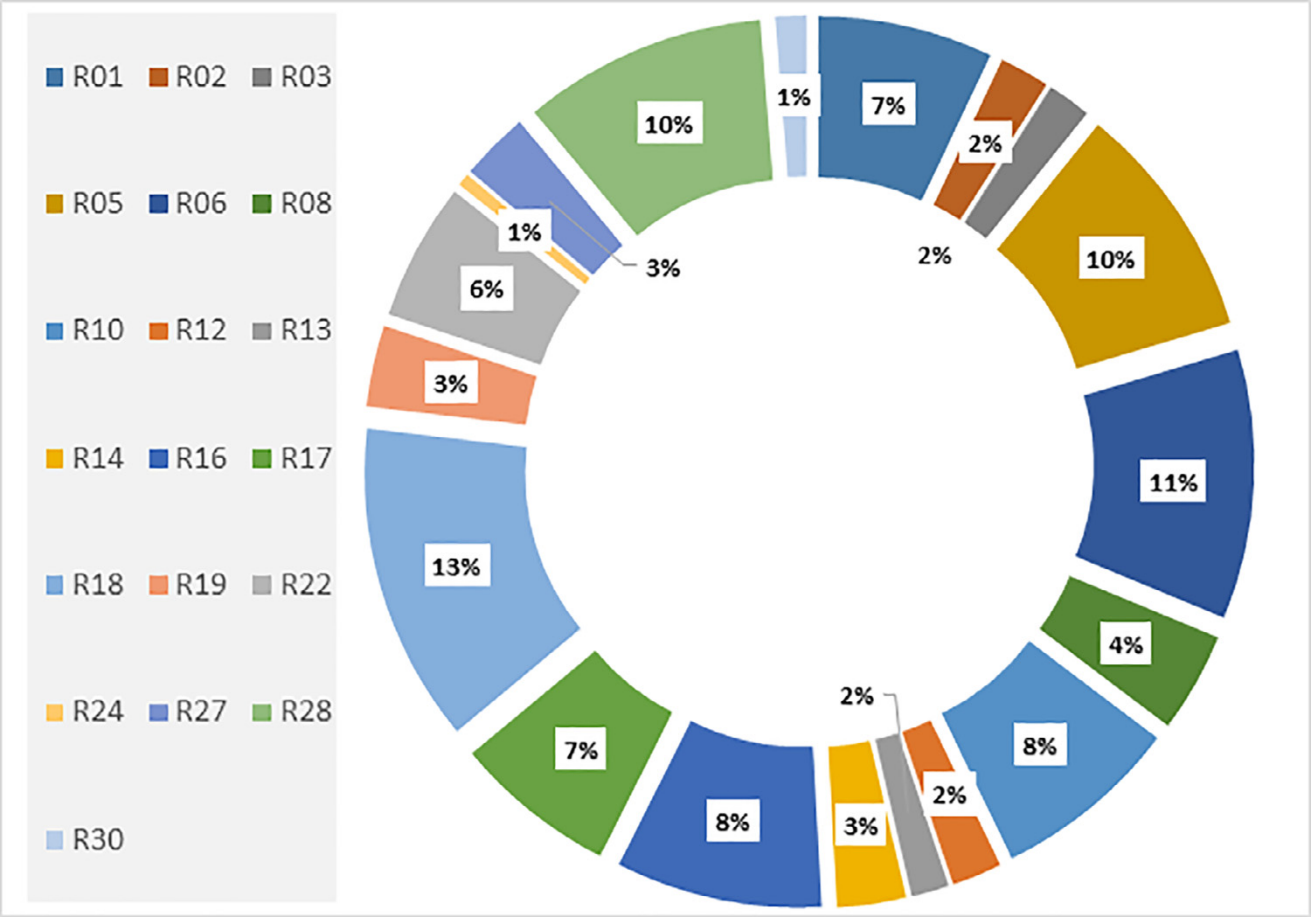
Distribution of imitation clips per reciter.

In terms of the distribution of imitation clips per reciters, we manage to collect imitations for 19 reciters out of the main 30 reciters as explored in Table 1 for further details on the reciters list and deployment procedures please see in [2], [3]. Nonetheless, the distribution of imitation clips per reciter is shown in Fig. 2; the majority of those covered are well-known to be the most influential and therefore have been imitated by many new learners.

**Table 1:** Data of 12 imitators and all reciters.

**SN**	**Rec. ID**	**I01**	**I02**	**I03**	**I04**	**I05**	**I06**	**I07**	**I08**	**I09**	**I10**	**I11**	**I12**	**Total**
1	R01					5					23			**28**
2	R02					6				2				**8**
3	R03					7								**7**
4	R04													**0**
5	R05	22	2				5		9					**38**
6	R06		2			3		36		2				**43**
7	R07													**0**
8	R08				7	4	3			2				**16**
9	R09													0
10	R10					11						10	9	**30**
11	R11													**0**
12	R12					8								**8**
13	R13				2					4				**6**
14	R14				9	2								**11**
15	R15													**0**
16	R16	10	2	4	8	5				4				**33**
17	R17			20	6									**26**
18	R18	14	4		14	5	5		6	3				**51**
19	R19				3	7				3				**13**
20	R20													**0**
21	R21													**0**
22	R22				15		4			3				**22**
23	R23													**0**
24	R24					2								**2**
25	R25													**0**
26	R26													**0**
27	R27					7				4				**11**
28	R28	20				4			2	13				**39**
29	R29													**0**
30	R30					5								**5**
**Total**		**66**	**10**	**24**	**64**	**81**	**17**	**36**	**17**	**40**	**23**	**10**	**9**	**397**

## 2 Experimental Design, Materials and Methods

There is a considerable gap when it comes to audio datasets when compared with the computer vision domain where large datasets such as MNIST^1^ and ImageNet^2^ became a baseline for researchers to evaluate their work, or text-based datasets on the rise including those dedicated for the Arabic language such as [1].

Its important to know that there are ten different known Quranic recitations or reading methods (Qira‘at). In each recitation, there are two famous narrations (Rewayah) that have been authenticated and narrated from qualified and elder licensed scientists (Sheikh) to their students [4]. The most popular recitation is that of Hafs Bin Suleiman, on the authority of Asim Al-Koufi, which is being recited in Arabia, Levant, Iraq, Egypt, India, Pakistan and Turkey.

An important aspect of creating this dataset was the careful selection of representative data to cover different countries within the same recitation method. We aimed to account for any subtle differences in reciters cantillations as a uniquely improvised melodic style which may be influenced by dialect language, cultures, maturity, and popularity of reciters. Nonetheless, the selected reciters here are identified as the most popular in their respective country of origin with great influence on new readers/learners who often start their training by imitating those popular reciters until they develop their own distinct style.

The curated audio samples averaged 10 seconds in length and for the actual deployment in our published experiments  [3] they were pre-processed by acoustic analysis (Seewave and tuneR packages), with an analyzed frequency range of 80-280 hz (human vocal range). The clips were then saved in WAV format, mono 16 bits to ensure uniformity. Additional tools were also used in our experiments and may also be utilized in further work including Adobe Audition, Wave Pad, Audacity Quick Player, and Switch.

## CRediT Author Statement

**Mohammed Lataifeh:** Conceptualization, Resources, Visualization, Writing - original draft. **Ashraf Elnagar:** Methodology, Data curation, Software, Writing - review & editing.

## Ethics Statement

This work was conducted in compliance with relevant ethical guidelines. The collected data clips were scrapped from the public domain for well-known reciters, these materials are typically shared as an Islamic Endowment disseminated over the web to be downloaded by audience interested in the subject matter. As for the imitators’ data where social media were used to survey the most popular imitators, all identifying materials for the collected imitators’ clips were anonymized and comply with data redistribution policies from the concerned platform.

## Declaration of Competing Interest

The authors declare that they have no known competing financial interests or personal relationships that could have appeared to influence the work reported in this paper.

## Footnotes

1 yann.lecun.com/exdb/mnist/ [Accessed Dec. 5, 2019]

2 http://www.image-net.org/ [Accessed Dec. 5, 2019]
